# Tolerability and Safety of Large-Volume Hyaluronidase-Facilitated Subcutaneous Immunoglobulin 10% Administered with or without Dose Ramp-Up: A Phase 1 Study in Healthy Participants

**DOI:** 10.1007/s10875-024-01742-5

**Published:** 2024-06-19

**Authors:** Zhaoyang Li, Andras Nagy, Dirk Lindner, Kim Duff, Enrique Garcia, Hakan Ay, Juan Carlos Rondon, Leman Yel

**Affiliations:** 1 Takeda Development Center Americas, Inc, Cambridge, MA USA; 2 Baxalta Innovations GmbH, a Takeda company, Vienna, Austria; 3 Clinical Pharmacology of Miami, LLC, Miami, FL USA; 4 University of California, Irvine, CA USA

**Keywords:** (4–6 should be Provided) Tolerability, Safety, Facilitated Subcutaneous Immunoglobulin (fSCIG), Dose ramp-up

## Abstract

**Purpose:**

Facilitated subcutaneous immunoglobulin (fSCIG; immune globulin infusion 10% [human] with recombinant human hyaluronidase [rHuPH20]) permits high-volume subcutaneous immunoglobulin (SCIG) infusion, shorter infusion times and reduced dosing frequency relative to conventional SCIG. It is initiated by gradually increasing infusion volumes over time (dose ramp-up) to achieve target dose level (TDL). Whether ramp-up strategies have tolerability or safety advantages over direct initiation at full TDL has not been evaluated clinically.

**Methods:**

This phase 1 open-label study assessed tolerability and safety of fSCIG 10% with accelerated or no ramp-up compared with conventional ramp-up in healthy adults (NCT04578535). Participants were assigned to one of the three ramp-up arms to achieve TDLs of 0.4 or 1.0 g/kg/infusion. The primary endpoint was the proportion of infusions completed without interruption or infusion rate reduction owing to treatment-emergent adverse events (TEAEs). Safety was assessed as a secondary endpoint.

**Results:**

Of 51 participants enrolled, 50 (98.0%) tolerated all fSCIG 10% infusions initiated (*n =* 174). Infusion rate was reduced in one participant owing to headache in the 0.4 g/kg/infusion conventional ramp-up arm. Study discontinuations were higher in the no ramp-up arm (70%) versus the conventional (0%) and accelerated (22%) arms at the 1.0 g/kg/infusion TDL. Safety outcomes did not substantially differ between treatment arms.

**Conclusion:**

The favorable tolerability and safety profiles of fSCIG 10% in healthy participants support initiating treatment with fSCIG 10% with accelerated ramp-up at TDLs up to 1.0 g/kg. Data support no ramp-up at TDLs close to 0.4 g/kg but additional data are needed for higher doses.

**Supplementary Information:**

The online version contains supplementary material available at 10.1007/s10875-024-01742-5.

## Introduction

Immunoglobulin (Ig) therapies originated in the 1950s for the treatment of Ig deficiencies and have since been further developed to address a variety of autoimmune and chronic inflammatory diseases [1, 2]. Igs have two broad therapeutic uses. In Ig replacement therapy (IgRT), Ig preparations derived from pooled donor plasma are administered to patients with inborn and acquired deficits in immunity, e.g., primary immunodeficiency diseases (PID; also known as inborn errors of immunity [3]), secondary immunodeficiency diseases, and hypogammaglobulinemia [4]. In some neuroinflammatory conditions, such as chronic inflammatory demyelinating polyradiculoneuropathy (CIDP) and multifocal motor neuropathy (MMN), Ig therapy can be used as an immunomodulator owing to its anti-inflammatory properties [5].

Ig therapies have two typical methods of delivery, each with associated benefits and limitations. Intravenous Ig (IVIG) allows for large infusion volumes to be administered once every 3–4 weeks, but requires venous access and the support of trained personnel [6–8]. Subcutaneous Ig (SCIG) provides greater flexibility for patients because no venous access is required, thereby reducing the need for clinic visits; however, conventional SCIG has a lower maximum volume of infusion and, consequently, requires more frequent dosing than IVIG. SCIG has fewer systemic side effects compared with IVIG, but it often induces minor localized, transient, infusion site reactions [6, 8].

HYQVIA/HyQvia (Baxalta US, Inc., a Takeda company, Lexington, MA, USA/Baxalta Innovations GmbH, a Takeda Company, Vienna, Austria [9, 10]) is a facilitated SCIG (fSCIG), administered as a two-component sequential infusion of recombinant human hyaluronidase (rHuPH20) followed by Ig 10% infusion within approximately 10 min. rHuPH20 depolymerizes hyaluronan in subcutaneous tissue, transiently increasing tissue permeability to Ig. This allows high-volume Ig administration (up to 600 mL/infusion site) into the subcutaneous tissue over a short time [11], and permits higher infusion rates and reduced dosing frequency compared with conventional SCIG [12].

Many patients initiating treatment with fSCIG 10% may be inexperienced in using the subcutaneous route of administration, and those transitioning from conventional SCIG may not have experienced the larger infusion volumes that fSCIG 10% can deliver. Dose ramp-up strategies incrementally increase infusion volumes to achieve target doses and help patients adapt to high-volume subcutaneous infusions. The following ramp-up schedule is included in the current US prescribing information for fSCIG 10% for the treatment of PID [9]: for a 4-week dosing interval, the target dose level (TDL) is achieved by starting at 25% of the TDL in Week 1, increasing to 50% in Week 2, 75% in Week 4, and 100% in Week 7 [9]. However, there is some evidence supporting the use of an accelerated ramp-up schedule [13], where the target dose is achieved over a shorter time frame (e.g., reaching the TDL at Week 5 rather than Week 7) with larger stepped increases in infusion volumes (e.g., 50% of TDL increments rather than 25% increments). However, to date, no clinical trials have been conducted that have evaluated the recommended ramp-up schedule versus accelerated ramp-up or versus treatment initiation without ramp-up (i.e., initiating treatment with the full TDL at Week 1). Achieving a therapeutic dose faster than with the recommended ramp-up schedule may provide benefits and convenience to patients, providing that these alternative schedules are well tolerated with favorable safety.

Studying healthy individuals allows for the examination of the tolerability and safety of investigational treatments without interference by concomitant conditions. Thus, any difference in tolerability or safety emerging between treatment arms would be expected to be due primarily to differences in dosing. The purpose of this phase 1 study was to assess the feasibility of initiating fSCIG 10% at the full TDL (i.e., no ramp-up) or using an accelerated ramp-up versus a conventional ramp-up, similar to that recommended in the fSCIG 10% prescribing information, to achieve two different TDLs: a lower TDL (0.4 g/kg/infusion) relevant to IgRT [9] or a higher TDL (1.0 g/kg/infusion) relevant to Ig use for neuroinflammatory conditions [5]. The primary objective was to assess the tolerability of fSCIG 10% across the three dosing schedules (conventional, accelerated, and no ramp-up) and the two TDLs (0.4 g/kg/infusion and 1.0 g/kg/infusion). The secondary objective was to assess the safety of fSCIG 10% (including immunogenicity against rHuPH20) across each of the three dosing schedules.

## Methods

### Study Design

**Fig. 1:**
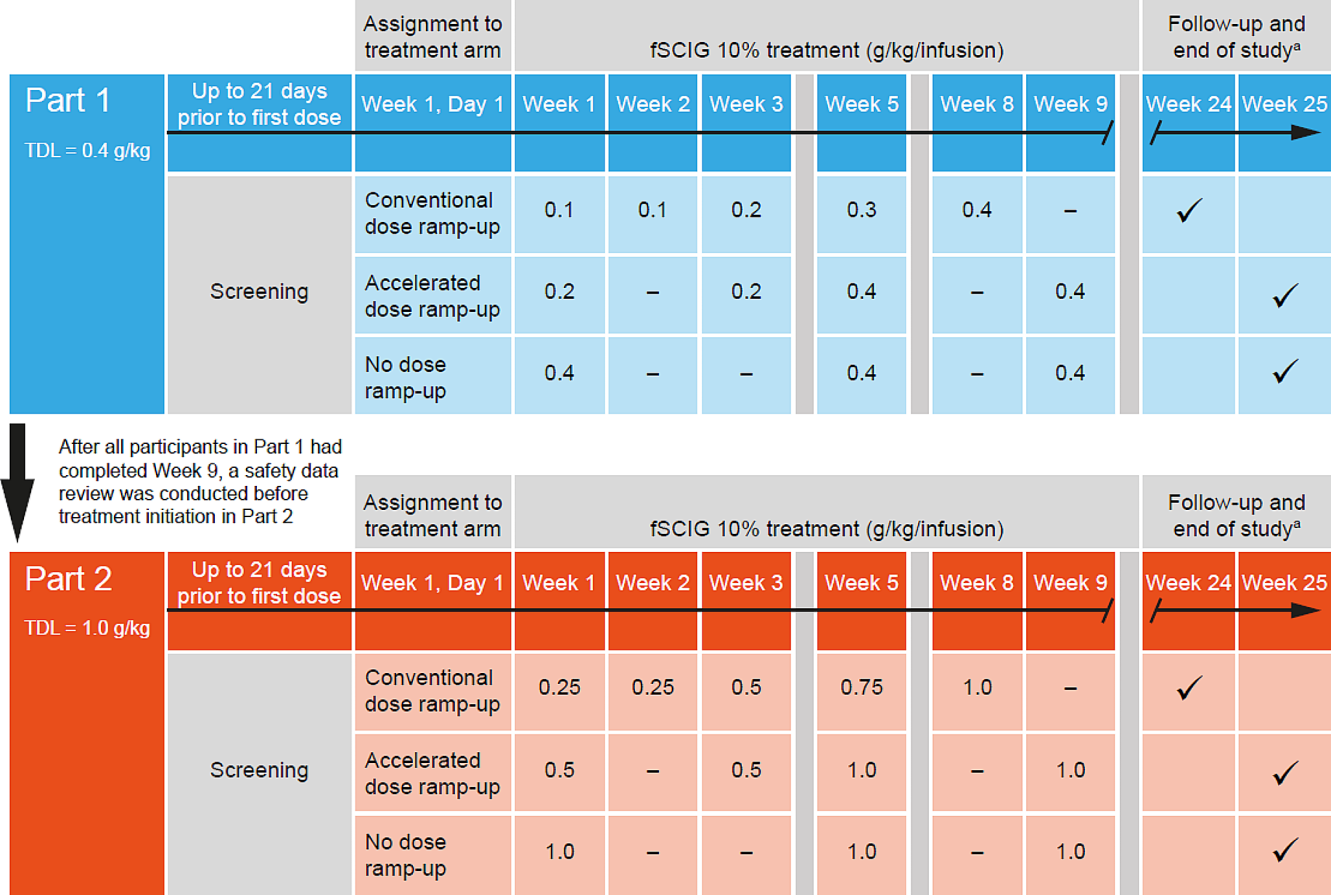
Study schematic. The conventional dose ramp-up schedule is 1 week longer than that recommended in the fSCIG 10% prescribing information with the inclusion of an additional dose in Week 1. This was done to match the schedule used in the ADVANCE-CIDP 1 study (NCT02549170) [11]. ^a^Time points at which follow-up and end of study occurred are shown by check marks in the Week 24 and Week 25 columns. Recombinant human hyaluronidase was administered prior to the immunoglobulin infusion at a dose of 80 U/g immunoglobulin or 0.5 mL/10 mL immunoglobulin. *fSCIG*, facilitated subcutaneous immunoglobulin; *TDL*, target dose level

This was a phase 1, open-label, single-center study (NCT04578535) of fSCIG 10% (HYQVIA/HyQvia [9, 10]) conducted in healthy participants in the USA, which took place from November 4, 2020 (first participant dosed with study drug) to March 2, 2022 (last participant completed the study). The study was conducted in two parts that ran sequentially (Part 1 followed by Part 2), each with three periods (Fig. 1). The parts differed in the TDL: Part 1 had a TDL of 0.4 g/kg/infusion (equivalent to a volume of 4 mL/kg/infusion), and Part 2 had a TDL of 1.0 g/kg/infusion (equivalent to 10 mL/kg/infusion). In each part, there was an initial screening period of up to 21 days prior to administration of the first dose of study treatment. This was followed by the study treatment period, which lasted until Week 8 or Week 9, as per the treatment arm schedules. The treatment arms were conventional dose ramp-up, accelerated ramp-up, and no ramp-up, which differed according to their dosing schedules. The conventional ramp-up schedule started at 25% of the TDL in Weeks 1 and 2, 50% of the TDL in Week 3, 75% of the TDL in Week 5, and the full TDL in Week 8. This ramp-up schedule was used in the ADVANCE-CIDP 1 study (NCT02549170) [11], and was intended to represent a schedule used to initiate a 4-weekly dosing regimen. The conventional ramp-up used in the current study differs from the schedule recommended in the fSCIG 10% prescribing information in that it includes two initial weekly doses at 25% of the TDL, rather than a single dose. The accelerated ramp-up schedule started at 50% of the TDL in Weeks 1 and 3, with the full TDL administered in Weeks 5 and 9. In the no ramp-up arm, the full TDL was administered at Weeks 1, 5, and 9. Across all treatment arms, each dose was administered on the first day of the week indicated. Upon completion of treatment in Weeks 8 or 9 of Part 1, a safety review team examined tolerability and safety data before commencement of Part 2. The follow-up period lasted up to 16 ± 1 weeks after the last infusion (Fig. 1).

fSCIG 10% infusions were administered at one or two infusion sites. If two sites were used, they were on opposite sides of the body. Maximum fSCIG 10% infusion volume (excluding rHuPH20 volume) was up to 600 mL/day at one site or up to 1200 mL/day at two sites. The rHuPH20 and Ig components were infused sequentially, starting with rHuPH20.

### Study Participants and Treatment Allocation

Participants were required to be: aged 19–50 years at the date of informed consent; male, or non-pregnant, non-breastfeeding females who agreed to comply with contraceptive requirements, or females of non-childbearing potential; determined to be healthy by the study investigator following a physical examination and assessment of medical records; and have a body mass index (BMI) of 18–30 kg/m^2^. A full list of eligibility criteria is provided in Supplementary Methods. The planned sample size for this study was 48 participants (eight in each of the six treatment arms, with a minimum of three participants in each BMI subgroup [i.e., 18 to < 25 kg/m^2^ and ≥ 25 to ≤ 30 kg/m^2^ in each treatment arm]). Each participant was assigned to only one treatment arm. Participant assignment was planned to follow a ratio of 1:1:1.

### Endpoints

The primary endpoint was the tolerability of fSCIG 10%. This was assessed as the proportion of participants who completed all initiated infusions without interruption, stoppage, or infusion rate reduction due to a treatment-emergent adverse event (TEAE) that began during the infusion, related to either the rHuPH20 or Ig components. One of the secondary endpoints was the safety of fSCIG 10% infusions. Throughout the study, participants were monitored with clinical laboratory measurements, physical examinations, vital sign measurements, and electrocardiograms. Safety was evaluated by the number of TEAEs and rates of TEAE per participant, per infusion, and per person-year. TEAEs were recorded according to whether they were: fSCIG-related or non-related; non-serious or serious; mild, moderate, or severe; local or systemic; infusion-associated (i.e., any TEAE that began during the infusion or within 24 h following infusion); or led to premature discontinuation from the study. Adverse reactions (ARs) and ARs of special interest were also recorded. The number of participants who developed any binding or neutralizing anti-rHuPH20 antibodies was recorded, and the proportion of participants with anti-rHuPH20 binding antibody titers ≥ 1:160 was summarized between treatment arms [14]. For the determination of binding anti-rHuPH20 antibodies, plasma samples were assayed using a bridging format electrochemiluminescence immunoassay method validated according to current guidance [15] and industry standards [16] at Eurofins Pharma Bioanalytics Services, St Charles, MO, USA. In the event that binding anti-rHuPH20 antibody titers ≥ 1:160 were detected, plasma samples were assessed for neutralizing anti-rHuPH20 antibodies using a validated hyaluronidase enzymatic assay (BioAgilytix, San Diego, CA, USA).

### Statistical Analysis

This study was not designed for statistical hypothesis testing; therefore, the sample size was not based on statistical considerations. All participants who received at least one dose of fSCIG 10% were included in the analysis. Tolerability and safety outcomes were compared between the conventional, accelerated, and no ramp-up arms for TDLs of 0.4 g/kg/infusion and 1.0 g/kg/infusion (i.e., study Part 1 and Part 2) separately.

### Ethics

This study was conducted in accordance with the International Council for Harmonisation Guideline for Good Clinical Practice. The study was approved by the IntegReview Institutional Review Board on May 26, 2020.

## Results

### Participant Disposition

**Fig. 2:**
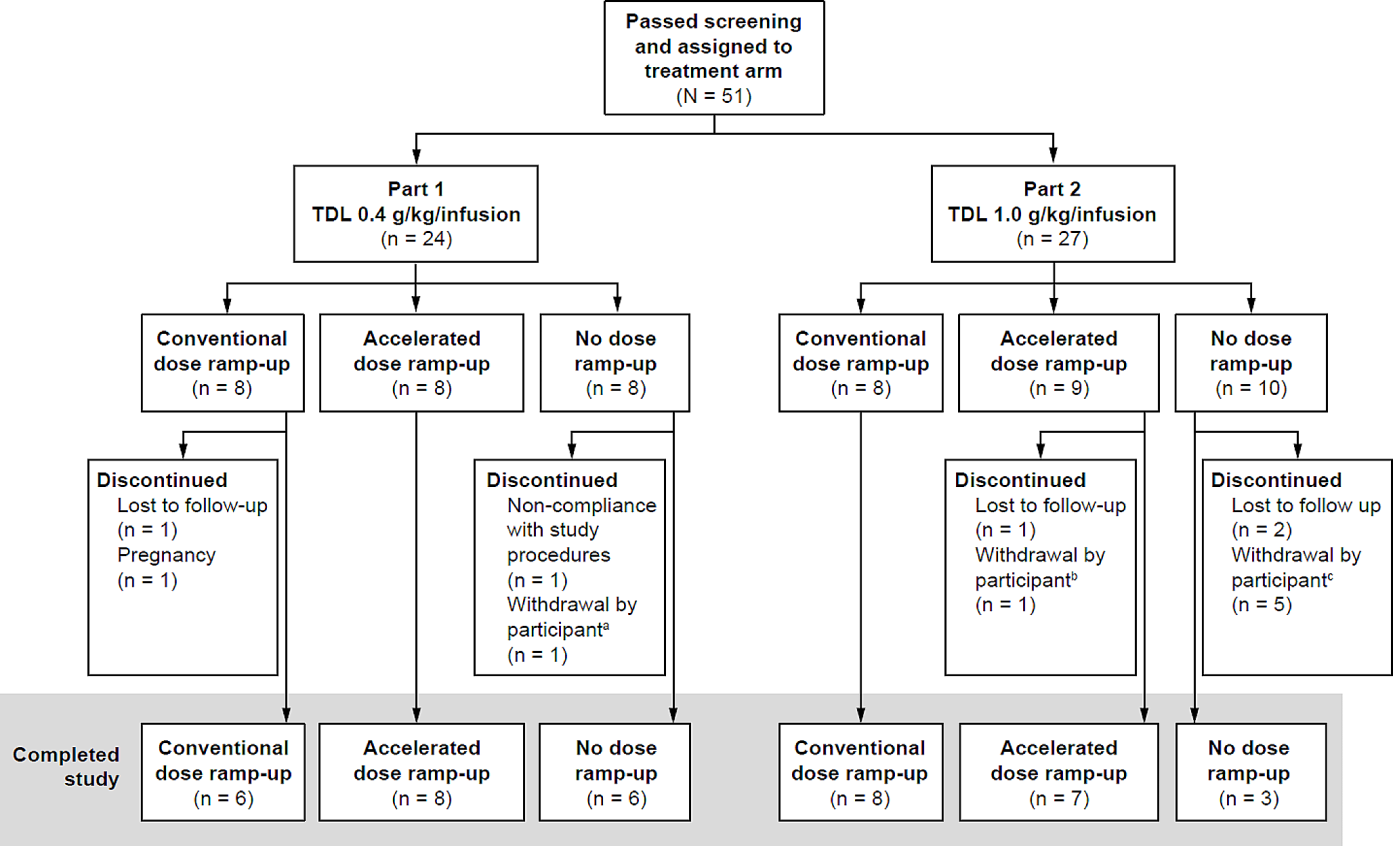
Participant disposition and discontinuations. ^a^The participant gave no specific reason for withdrawal. ^b^The participant was unable to complete the study due to their work schedule. ^c^Reasons for withdrawal were not related to the occurrence of fSCIG-related adverse events. *TDL*, target dose level

A total of 51 participants completed screening and were enrolled into the study, of whom 38 (74.5%) completed the study and 13 (25.5%) discontinued (Fig. 2). This sample comprised the prespecified number of 48 participants as well as a further three participants who were included as replacements for some of the participants who discontinued participation in the accelerated (*n =* 1) and no dose ramp-up (*n =* 2) arms at the 1.0 g/kg/infusion TDL. Replacements were included to ensure that at least six participants completed each treatment arm. When it became apparent that discontinuations from the study were more frequent in the no ramp-up arm at the 1.0 g/kg/infusion TDL, the decision was made to make no further replacements. This led to a minor imbalance in the planned 1:1:1 assignment to treatment arms at this TDL. Baseline characteristics of the study population are provided in Table 1.

**Table 1:** Participant demographics by treatment arm

	Part 1 TDL 0.4 g/kg/infusion (4 mL/kg/infusion)^a^			Part 2 TDL 1.0 g/kg/infusion (10 mL/kg/infusion)^a^			Overall (*N* = 51)
Characteristic	Conventional dose ramp-up (*n =* 8)	Accelerated dose ramp-up (*n =* 8)	No dose ramp-up (*n =* 8)	Conventional dose ramp-up (*n =* 8)	Accelerated dose ramp-up (*n =* 9)	No dose ramp-up (*n =* 10)	
Age, years							
Mean (SD)	28.9 (8.04)	40.4 (5.01)	36.8 (8.58)	34.4 (6.44)	36.4 (7.88)	34.0 (10.07)	35.1 (8.28)
Sex, *n* (%)							
Female	7 (87.5)	4 (50.0)	4 (50.0)	5 (62.5)	5 (55.6)	4 (40.0)	29 (56.9)
Ethnicity, *n* (%)							
Hispanic or Latino	8 (100)	6 (75.0)	6 (75.0)	8 (100)	9 (100)	10 (100)	47 (92.2)
Race, *n* (%)							
Black or African American	4 (50.0)	4 (50.0)	2 (25.0)	2 (25.0)	3 (33.3)	2 (20.0)	17 (33.3)
White	4 (50.0)	4 (50.0)	6 (75.0)	6 (75.0)	6 (66.7)	8 (80.0)	34 (66.7)
BMI, *n* (%)							
18 to < 25 kg/m^2^	4 (50.0)	3 (37.5)	3 (37.5)	4 (50.0)	4 (44.4)	4 (40.0)	22 (43.1)
25 to ≤ 30 kg/m^2^	4 (50.0)	5 (62.5)	5 (62.5)	4 (50.0)	5 (55.6)	6 (60.0)	29 (56.9)

^a^Recombinant human hyaluronidase was administered prior to the immunoglobulin infusion at a dose of 80 U/g immunoglobulin or 0.5 mL/10 mL immunoglobulin. *BMI*, body mass index; *SD*, standard deviation; *TDL*, target dose level

At the 0.4 g/kg/infusion TDL, all participants in the accelerated ramp-up arm completed the study, whereas two participants in the conventional ramp-up arm and two participants in the no ramp-up arm discontinued. At the 1.0 g/kg/infusion TDL, discontinuations were higher in the no ramp-up arm (*n =* 7) compared with the conventional (*n =* 0) or accelerated (*n =* 2) ramp-up arms. Reasons for discontinuation are shown in Fig. 2.

### Tolerability

At the 0.4 g/kg/infusion TDL, all participants in the accelerated and no ramp-up arms completed all initiated infusions, and in the conventional ramp-up arm, seven out of eight participants (87.5%) tolerated all infusions initiated (Supplementary Table S1). The exception was one participant who experienced a TEAE of headache approximately 1 h after the start of infusion of the Ig component of fSCIG 10% when administered at the full TDL in Week 8. In response, the infusion rate was reduced, after which the headache resolved in 40 min. The participant received the complete infusion and tolerated all other initiated infusions. At the 1.0 g/kg/infusion TDL, all participants in all treatment arms tolerated all initiated infusions (Supplementary Table S1).

### Safety

All participants experienced TEAEs during the study, with a total of 422 events reported (402 local, 20 systemic) (Table 2, Supplementary Table S2). Of these, all were considered as ARs or suspected ARs of special interest and all were classified as non-serious, with no moderate or severe TEAEs reported (all TEAEs were mild). The local TEAEs occurring at infusion sites were swelling (100% of participants), erythema (80.4%), pain (68.6%), pruritus (52.9%), and extravasation (leaking at the infusion site, 2.0%) (Supplementary Table S2). The 1.0 g/kg/infusion TDL no ramp-up group had the highest incidence of systemic TEAEs (including headache, pyrexia, and dizziness), with 50.0% of participants experiencing a total of 11 events, of which six were considered related to treatment.

**Table 2:** Summary of TEAEs across treatment arms

Category, *n* (%) events recorded	Part 1 TDL 0.4 g/kg/infusion (4 mL/kg/infusion)^a^			Part 2 TDL 1.0 g/kg/infusion (10 mL/kg/infusion)^a^			Overall (*N* = 51)
	Conventional dose ramp-up (*n =* 8)	Accelerated dose ramp-up (*n =* 8)	No dose ramp-up (*n =* 8)	Conventional dose ramp-up (*n =* 8)	Accelerated dose ramp-up (*n =* 9)	No dose ramp-up (*n =* 10)	
Any TEAE^b^	8 (100) 65	8 (100) 69	8 (100) 50	8 (100) 82	9 (100) 91	10 (100) 65	51 (100) 422
Severe	0	0	0	0	0	0	0
TEAEs leading to death	0	0	0	0	0	0	0
Serious	0	0	0	0	0	0	0
Non-serious	8 (100) 65	8 (100) 69	8 (100) 50	8 (100) 82	9 (100) 91	10 (100) 65	51 (100) 422
fSCIG-related	8 (100) 65	8 (100) 67	8 (100) 50	8 (100) 82	9 (100) 91	10 (100) 60	51 (100) 415
fSCIG-non-related	0	2 (25.0) 2	0	0	0	3 (30.0) 5	5 (9.8) 7
Local	8 (100) 62	8 (100) 67	8 (100) 48	8 (100) 81	9 (100) 90	10 (100) 54	51 (100) 402
Systemic	1 (12.5) 3	2 (25.0) 2	2 (25.0) 2	1 (12.5) 1	1 (11.1) 1	5 (50.0) 11	12 (23.5) 20
TEAEs leading to discontinuation	0	0	0	0	0	0	0
Infusion-associated	8 (100) 65	8 (100) 69	8 (100) 49	8 (100) 82	9 (100) 91	10 (100) 57	51 (100) 413
Temporally associated	8 (100) 65	8 (100) 69	8 (100) 49	8 (100) 82	9 (100) 91	10 (100) 64	51 (100) 420
AR plus suspected AR of special interest	8 (100) 65	8 (100) 69	8 (100) 50	8 (100) 82	9 (100) 91	10 (100) 65	51 (100) 422

^a^Recombinant human hyaluronidase was administered prior to the immunoglobulin infusion at a dose of 80 U/g immunoglobulin or 0.5 mL/10 mL immunoglobulin. ^b^All TEAEs experienced were mild in severity

*AR*, adverse reaction; *fSCIG*, facilitated subcutaneous immunoglobulin; *TDL*, target dose level; *TEAE*, treatment-emergent adverse event

**Fig. 3:**
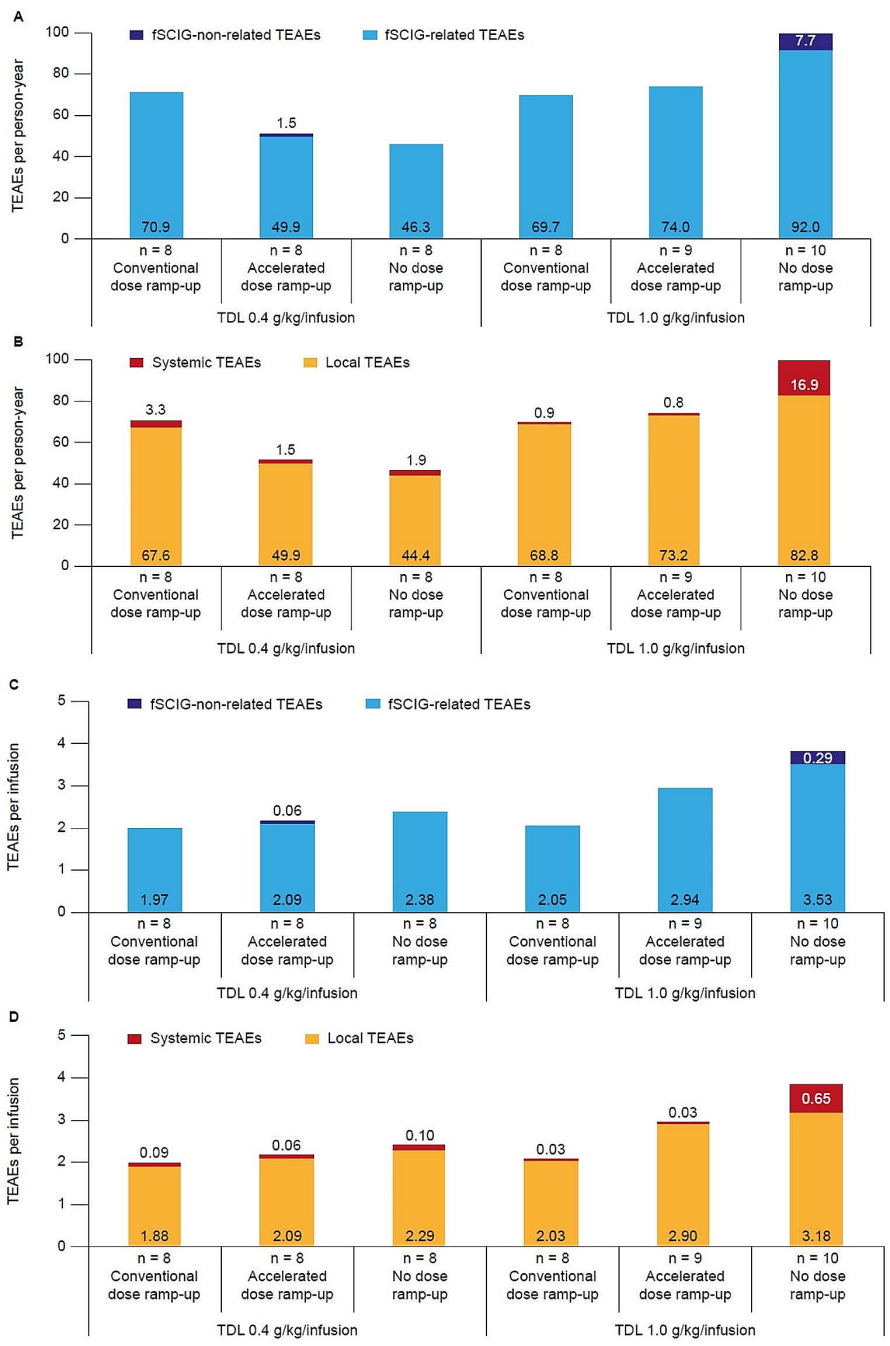
Rates of fSCIG-related versus fSCIG-non-related TEAEs and local versus systemic TEAEs per person-year (panels **A** and **B**, respectively) and per infusion (panels **C** and **D**, respectively). *fSCIG*, facilitated subcutaneous immunoglobulin; *TDL*, target dose level; *TEAE*, treatment-emergent adverse event

All participants experienced fSCIG-related TEAEs (415 events) and there were seven fSCIG-non-related events (two in the 0.4 g/kg/infusion TDL accelerated ramp-up arm and five in the 1.0 g/kg/infusion TDL no ramp-up arm) (Table 2). The rates of fSCIG-non-related versus fSCIG-related TEAEs and systemic versus local TEAEs per person-year and per infusion are shown in Fig. 3. At the 0.4 g/kg/infusion TDL, the rate of any TEAE (per person-year) was highest in the conventional ramp-up arm (70.9) compared with the accelerated (51.4) and no ramp-up (46.3) arms (Fig. 3A and B). This difference was driven by a higher rate of local TEAEs in the conventional ramp-up arm (67.6 per person-year vs. 49.9 in the accelerated ramp-up arm and 44.4 in the no ramp-up arm), while the rate of systemic TEAEs was similar between groups (3.3, 1.5, and 1.9 per person-year in the conventional, accelerated, and no ramp-up arms, respectively) (Fig. 3B). However, it should be noted that the number of infusions administered was not equal between treatment arms (Fig. 1). The corresponding rates of any TEAE per infusion were 2.0 with conventional ramp-up, 2.2 with accelerated ramp-up, and 2.4 with no ramp-up (Fig. 3C and D).

At the 1.0 g/kg/infusion TDL, the rate of any TEAE (per person-year) was highest in the no ramp-up arm (99.7) compared with the conventional (69.7) and accelerated (74.0) ramp-up arms (Fig. 3A and B). Per infusion, the rates were 3.8 with no ramp-up, 2.1 with conventional ramp-up, and 2.9 with accelerated ramp-up (Fig. 3C and D). For this TDL, there was a higher rate (per person-year) of systemic TEAEs in the no ramp-up arm (16.9) compared with the other treatment arms (0.9 and 0.8, for conventional and accelerated ramp-up arms, respectively) (Fig. 3B).

Binding anti-rHuPH20 antibodies were detected in participants in all treatment arms at both TDLs at various time points in the study (Supplementary Table S3). No participants developed binding anti-rHuPH20 antibody responses (titers ≥ 1:160). Therefore, no further testing was conducted to assess neutralizing anti-rHuPH20 antibodies.

## Discussion

A dose ramp-up schedule is currently recommended for the initiation of fSCIG 10% treatment in patients with PID [9]. A similar conventional ramp-up schedule has also been included in the ADVANCE-CIDP 1 clinical trial that assessed the efficacy and safety of fSCIG 10% for the treatment of CIDP (NCT02549170) [11]. The primary objective of the present study was to understand whether accelerated ramp-up or treatment initiation at the full target dose are suitable alternatives to conventional ramp-up. We demonstrated that fSCIG 10% was well tolerated at both the 0.4 g/kg/infusion and 1.0 g/kg/infusion TDLs (equivalent to volumes of 4 mL/kg/infusion and 10 mL/kg/infusion, respectively), regardless of whether treatment was administered via conventional or accelerated ramp-up schedules or by direct initiation at the low TDL with no ramp-up in healthy participants.

Despite all participants experiencing TEAEs related to fSCIG 10% infusions, the majority of these were mild and local events, specifically swelling, erythema, pain, and pruritus at the infusion site. The overall incidence of systemic TEAEs was low across treatment arms, and the highest was observed in the no ramp-up arm at the 1.0 g/kg/infusion TDL. While these systemic events were mild in severity, this observation may indicate that a ramp-up approach at this higher TDL is more appropriate. Although the current data cannot differentiate between the two components of fSCIG 10% to determine if TEAEs were related to either Ig or rHuPH20, the contribution of rHuPH20 is believed to be minimal. Firstly, previous research has found no evidence of systemic exposure to rHuPH20 nor any discernible rHuPH20 activity in lymph or plasma as a function of time in animal models [17]. Secondly, the volume of rHuPH20 is small relative to the volume of Ig administered (0.5 mL/10 mL Ig), limiting the impact of its volume on local tolerability relative to Ig. rHuPH20 functions as a permeation enhancer and acts locally and temporally. Even if, to a limited extent, it is absorbed into systemic circulation, it would be rapidly cleared given its reported half-life of approximately 10 min in plasma, as demonstrated in a pharmacokinetic study of intravenously administered rHuPH20 [18]. Overall safety outcomes did not substantially differ between ramp-up and no ramp-up treatment arms, which were approximately BMI-matched owing to BMI-based stratified treatment allocation. Local infusion site TEAEs such as swelling are likely to occur with each infusion regardless of the infusion volume administered. This may explain why the rates of TEAEs per person-year at the 0.4 g/kg/infusion TDL are slightly elevated in the conventional ramp-up arm, which involved the most infusions, versus the accelerated ramp-up arm, which involved fewer infusions, followed by the no ramp-up arm, which involved the fewest infusions. However, at the 1.0 g/kg/infusion TDL, TEAEs per person-year do not follow this pattern, with the highest rate of TEAEs across treatment arms being in the no ramp-up arm. This, and the higher rate of participant discontinuation from the study in the no ramp-up group at the 1.0 g/kg/infusion TDL, may provide indirect evidence of improved tolerability with ramp-up dosing at this higher dose. At the 0.4 g/kg/infusion TDL, the rate of participant discontinuation from the study across treatment arms did not show a similar differentiation. Our findings are in accordance with a retrospective evaluation of alternative dosing regimens used in real-world clinical practice, which found that the use of accelerated ramp-up schedules was not associated with an increase in adverse events in patients with PID relative to conventional ramp-up schedules [13]. Another real-world study of 54 patients receiving fSCIG 10% showed that there were no severe TEAEs associated with an accelerated ramp-up schedule, and systemic TEAEs were rare [19]. Across all treatment arms, no participants had binding anti-rHuPH20 antibody titers ≥ 1:160, suggesting that there was no heightened immune response to the rHuPH20 component of fSCIG 10% infusions in immunocompetent individuals. Titers of anti-rHuPH20 antibodies < 1:160 are the result of passive transfer of rHuPH20-reactive antibodies contained in the therapeutic agent [14].

When using fSCIG 10% for IgRT at a TDL comparable with the lower TDL evaluated here [9], our findings suggest that flexibility in the dosing strategy can be employed at the discretion of clinicians and depending on the needs of individual patients. For immunomodulatory use of Igs, where doses needed may typically exceed the high TDL tested in this study [5], fSCIG 10% ramp-up strategies may be more suitable than direct initiation at 100% of the TDL, although an accelerated ramp-up may be considered. However, this is based on indirect evidence: namely, the higher rate of participant discontinuation from the study in addition to the higher rate of mild TEAEs seen in this small number of healthy participants without ramp-up. While these discontinuations after direct initiation at 100% of the TDL may be indicative of tolerability issues for healthy individuals who are inexperienced with large-volume subcutaneous infusions, this finding may not be fully representative of patients who have previously experienced SCIG dosing owing to the much larger infusion volumes of SCIG therapies compared with other subcutaneous therapies (for example, monoclonal antibodies). Given that fSCIG 10% is presently only approved for IgRT in patients with PID in the USA [9], the opportunities to examine ramp-up schedules in patients receiving Ig for other indications/diseases have hitherto been limited. A retrospective study using medical records of patients with PID in the USA showed that, in clinical practice, the rate of treatment discontinuation for off-label accelerated ramp-up schedules was low (1 in 25 patients, 4%) [13]. Although not directly comparable, this is a lower rate of treatment discontinuation than that seen for the lower TDL in our study (17%); this may be partly due to the perceived differences in the benefit of treatment between study samples. Currently there are no real-world data for off-label alternative ramp-up schedules at higher TDLs (≥ 1 g/kg/infusion) to allow us to compare with the rates of study discontinuations in the accelerated (22%) and no dose (70%) ramp-up arms reported here. Patients treated in the real world may be more willing to persist with ramp-up in the hope of achieving therapeutic benefits, unlike healthy participants in a clinical trial setting who will not gain therapeutic benefit from treatment.

As with any phase 1 study with healthy participants, the relevance of these findings may be limited when extrapolated to patients, owing to the disease state and concomitant conditions unlikely to be present in the study sample. It would be beneficial to replicate these findings in a larger number of patients with the indicated diseases, especially at higher TDLs. Nevertheless, this study has demonstrated that the tolerability and safety of Ig therapies in healthy participants is a valid and reasonable model which can be considered for future investigations of new Ig therapies.

## Conclusions

The majority of fSCIG 10% infusions at doses up to 1.0 g/kg/infusion with and without dose ramp-up were generally well tolerated with a favorable safety profile, and with few systemic side effects. For IgRT and immunomodulatory indications that require target doses up to 1.0 g/kg/infusion, a flexible approach to treatment initiation can be adopted. For patients who require fSCIG 10% doses of 1.0 g/kg/infusion or higher, it should be at the discretion of the individual treating physician as to whether to employ conventional or accelerated ramp-up approaches.

### Electronic Supplementary Material

Below is the link to the electronic supplementary material.

Supplementary Material 1

## Data Availability

The data sets, including the redacted study protocol, redacted statistical analysis plan, and individual participant data supporting the results reported in this article will be made available within 3 months from initial request to researchers who provide a methodologically sound proposal. The data will be provided after its de-identification, in compliance with applicable privacy laws, data protection, and requirements for consent and anonymization.
