# Supplementary material for: Tolerability and Safety of Large-Volume Hyaluronidase-Facilitated Subcutaneous Immunoglobulin 10% Administered with or without Dose Ramp-Up: A Phase 1 Study in Healthy Participants
Source: J Clin Immunol. 2024 Jun 19;44(7):148. doi: 10.1007/s10875-024-01742-5 (PMC11186899; doi:10.1007/s10875-024-01742-5)
Supplement: Supplementary file 1 — Supplementary Material 1 [file 10875_2024_1742_MOESM1_ESM.docx]

# Supplementary material

# Tolerability and Safety of Large-Volume Hyaluronidase-Facilitated Subcutaneous Immunoglobulin 10% Administered With or Without Dose Ramp-Up: A Phase 1 Study in Healthy Participants

*Journal of Clinical Immunology*

Zhaoyang Li^1^ • Andras Nagy^2^ • Dirk Lindner^1^ • Kim Duff^1^ • Enrique Garcia^1^ • Hakan Ay^1^ • Juan Carlos Rondon^3^ • Leman Yel^1*4^

*At time of study

Corresponding author:

Zhaoyang Li

zhaoyang.li@takeda.com

1 Takeda Development Center Americas, Inc., Cambridge, MA, USA

2 Baxalta Innovations GmbH, a Takeda company, Vienna, Austria

3 Clinical Pharmacology of Miami, LLC, Miami, FL, USA

4 University of California, Irvine, CA, USA

## Supplementary methods

### Study eligibility criteria

Inclusion criteria

1. An understanding, ability, and willingness to comply fully with study procedures and restrictions.
2. Ability to voluntarily provide written, signed, and dated informed consent (personally or via a legally authorized representative) to participate in the study.
3. Aged 19–50 years, inclusive, at the time of informed consent. The date of signature of the informed consent was defined as the beginning of the screening period. This inclusion criterion was only assessed at the first screening visit.
4. Male, or non-pregnant, non-breastfeeding female who agreed to comply with any applicable contraceptive requirements of the protocol, or females of non-childbearing potential.
5. Must have been considered healthy. Healthy status was determined by the investigator on the basis of screening evaluations, and was defined by the absence of evidence of any active or chronic disease following a detailed medical and surgical history, a complete physical examination including vital signs, a 12-lead electrocardiogram, hematology, blood chemistry, and urinalysis.
6. A BMI between 18.0 and 30.0 kg/m², inclusive.

Exclusion criteria

1. Any current or relevant history of medical (e.g., hematological, hepatic, respiratory, cardiovascular, renal, or neurological) or psychiatric conditions, which by judgment of the investigator might have compromised the safety of the participant or integrity of the study, interfered with participation in the trial, and compromised the trial objectives or any condition that presented undue risk from the investigational product (IP) or procedures. Note: participants on stable doses of hormone replacements (e.g., thyroid hormone replacement) or oral contraceptives were permitted to participate in the study.
2. Clinically significant cardiac conditions, including – but not limited to – uncontrolled hypertension, myocardial infarction, unstable coronary artery disease, and clinically significant arrythmias and conduction disorders.
3. Known or suspected intolerance or hypersensitivity to the IP(s), closely related compounds, or any of the stated ingredients (e.g., human immunoglobulin, hyaluronidase, albumin).
4. Known history of hypersensitivity or severe allergic reactions (e.g., urticaria, breathing difficulty, severe hypotension, or anaphylaxis) following administration of blood or blood components.
5. Significant illness, as judged by the investigator, within 30 days of the first dose of IP.
6. Known history of alcohol or other substance abuse within the last year.
7. Donation of blood within 60 days or blood products (e.g., plasma or platelets) within 2 weeks prior to receiving the first dose of IP.
8. Participants were excluded if any of the following laboratory parameters met the criteria below:
   1. Hemoglobin < 11 g/dL
   2. Absolute neutrophil count ≤ 1500/mm^3^ and platelet count ≤ 100,000/mm^3^
   3. Liver function: alanine aminotransferase ≥ 2.5 × upper limit of normal (ULN), aspartate aminotransferase ≥ 2.5 × ULN, alkaline phosphatase ≥ 1.5 × ULN, or total bilirubin ≥ 1.5 mg/dL
   4. Renal function: creatinine clearance ≤ 60 mL/min based on Cockcroft–Gault equation
   5. Coagulation tests: activated partial thromboplastin time > 1.2 × ULN; international normalized ratio > 1.2
   6. Participants were excluded if any other laboratory values were outside the reference range and were clinically significant as per the investigator’s judgment.
9. Within 30 days prior to the first dose of IP:
   1. Had participated in another clinical study involving immunoglobulin products within 12 months of screening
   2. Had used an IP (or 5 half-lives, whichever is longer)
   3. Had been enrolled in a clinical study (including vaccine studies or had been vaccinated with an approved product) that, in the investigator’s opinion, may have impacted this study. Participants who received any vaccine (including live attenuated vaccines) during the last 30 days before dosing were excluded. No live attenuated virus vaccines were allowed during the study until the end of the follow-up period
   4. Had any substantial changes in eating habits, as assessed by the investigator.
10. Confirmed systolic blood pressure > 139 mmHg or < 89 mmHg and diastolic blood pressure > 89 mmHg or < 49 mmHg.
11. A positive screen for alcohol or drugs of abuse at screening or day -1.
12. A positive human immunodeficiency virus, hepatitis C virus, or ongoing/active hepatitis B virus infection at screening. Participants with immunity to hepatitis B from either active vaccination or from previous natural infection were eligible to participate in the study.
13. Smoked more than 5 cigarettes or equivalent per day, or unable to stop smoking during confinement in the clinical research unit.
14. Severe dermatitis or anatomical abnormality that would have interfered with facilitated subcutaneous immunoglobulin administration or endpoint assessments. Note: the skin at the administration site should not have been covered by tattoos.
15. Current use of any herbal or homeopathic preparations was not permitted.
16. Unable or unwilling to discontinue antihistamines or medications with antihistamine properties, sedatives, anxiolytics, systemic or topical steroids, or antibiotics on any area below the chest for a minimum of 48 hours prior to each infusion visit and through 72 hours after the last infusion.
17. Current or relevant history of hypercoagulable conditions (e.g., protein C, protein S, and antithrombin III deficiency), thrombotic/thromboembolic events, or venous thrombosis.

## Table S1 Number and proportions of participants who tolerated fSCIG 10% infusions and components overall and by BMI category

|  | Part 1  TDL 0.4 g/kg/infusion | | | Part 2  TDL 1.0 g/kg/infusion | | | Overall  (*N*=51) |
| --- | --- | --- | --- | --- | --- | --- | --- |
| Category, *n* (%) | Conventional dose  ramp-up  (*n=*8) | Accelerated dose  ramp-up  (*n=*8) | No dose  ramp-up  (*n=*8) | Conventional dose  ramp-up  (*n=*8) | Accelerated dose  ramp-up  (*n=*9) | No dose ramp-up (*n=*10) |  |
| fSCIG 10% infusion  All participants | 7 (87.5) | 8 (100) | 8 (100) | 8 (100) | 9 (100) | 10 (100) | 50 (98.0) |
| BMI 18 to < 25 kg/m^2^ | 4 (100) | 3 (100) | 3 (100) | 4 (100) | 4 (100) | 4 (100) | 22 (100) |
| BMI 25 to ≤ 30 kg/m^2^ | 3 (75) | 5 (100) | 5 (100) | 4 (100) | 5 (100) | 6 (100) | 28 (96.6) |
| rHuPH20 component  All participants | 8 (100) | 8 (100) | 8 (100) | 8 (100) | 9 (100) | 10 (100) | 51 (100) |
| BMI 18 to < 25 kg/m^2^ | 4 (100) | 3 (100) | 3 (100) | 4 (100) | 4 (100) | 4 (100) | 22 (100) |
| BMI 25 to ≤ 30 kg/m^2^ | 4 (100) | 5 (100) | 5 (100) | 4 (100) | 5 (100) | 6 (100) | 29 (100) |
| Ig 10% component  All participants | 7 (87.5) | 8 (100) | 8 (100) | 8 (100) | 9 (100) | 10 (100) | 50 (98.0) |
| BMI 18 to < 25 kg/m^2^ | 4 (100) | 3 (100) | 3 (100) | 4 (100) | 4 (100) | 4 (100) | 22 (100) |
| BMI 25 to ≤ 30 kg/m^2^ | 3 (75) | 5 (100) | 5 (100) | 4 (100) | 5 (100) | 6 (100) | 28 (96.6) |

*BMI*, body mass index; *fSCIG*, facilitated subcutaneous immunoglobulin; *Ig*, immunoglobulin; *rHuPH20*, recombinant human hyaluronidase; *TDL*, target dose level

## Table S2 Types of local and systemic treatment-emergent adverse events by treatment arm

| **Category,** *n* (%)  events recorded | **Part 1**  **TDL 0.4 g/kg/infusion** | | | **Part 2**  **TDL 1.0 g/kg/infusion** | | | **Overall**  **(*N*=51)** |
| --- | --- | --- | --- | --- | --- | --- | --- |
|  | **Conventional dose  ramp-up**  **(*n=*8)** | **Accelerated dose  ramp-up**  **(*n=*8)** | **No dose  ramp-up**  **(*n=*8)** | **Conventional dose  ramp-up**  **(*n=*8)** | **Accelerated dose  ramp-up**  **(*n=*9)** | **No dose ramp-up (*n=*10)** |  |
| **Local**  Swelling  Erythema  Pain  Pruritus  Extravasation | **8 (100) 62**  8 (100) 26  5 (62.5) 18  3 (37.5) 5  4 (50.0) 13  0 | **8 (100) 67**  8 (100) 30  5 (62.5) 16  6 (75.0) 11  7 (87.5) 10  0 | **8 (100) 48**  8 (100) 21  6 (75.0) 12  5 (62.5) 11  3 (37.5) 4  0 | **8 (100) 81**  8 (100) 39  6 (75.0) 26  5 (62.5) 10  3 (37.5) 6  0 | **9 (100) 90**  9 (100) 32  9 (100) 27  9 (100) 18  5 (55.6) 12  1 (11.1) 1 | **10 (100) 54**  10 (100) 19  10 (100) 17  7 (70.0) 12  5 (50.0) 6  0 | **51 (100) 402**  51 (100) 167  41 (80.4) 116  35 (68.6) 67  27 (52.9) 51  1 (2.0) 1 |
| **Systemic**  Headache  Pyrexia  Dizziness  Nausea  Hypotension  Diarrhea  Vomiting  Chills  Pain  Systolic blood pressure decreased | **1 (12.5) 3**  1 (12.5) 1  0  0  1 (12.5) 1  0  0  0  1 (12.5) 1  0  0 | **2 (25.0) 2**  1 (12.5) 1  0  1 (12.5) 1  0  0  0  0  0  0  0 | **2 (25.0) 2**  2 (25.0) 2  0  0  0  0  0  0  0  0  0 | **1 (12.5) 1**  0  0  0  0  0  0  0  0  0  1 (12.5) 1 | **1 (11.1) 1**  0  0  0  1 (11.1) 1  0  0  0  0  0  0 | **5 (50.0) 11**  2 (20.0) 2  3 (30.0) 3  1 (10.0) 1  0  2 (20.0) 2  1 (10.0) 1  1 (10.0) 1  0  1 (10.0) 1  0 | **12 (23.5) 20**  6 (11.8) 6  3 (5.9) 3  2 (3.9) 2  2 (3.9) 2  2 (3.9) 2  1 (2.0) 1  1 (2.0) 1  1 (2.0) 1  1 (2.0) 1  1 (2.0) 1 |

*TDL*, target dose level

## Table S3 Binding and neutralizing anti-rHuPH20 antibodies

| **Timepoint**  **Anti-rHuPH20 antibody assay,** *n* (%) | **Part 1**  **TDL 0.4 g/kg/infusion** | | | **Part 2**  **TDL 1.0 g/kg/infusion** | | |
| --- | --- | --- | --- | --- | --- | --- |
|  | **Conventional dose  ramp-up**  **(*n=*8)** | **Accelerated dose  ramp-up**  **(*n=*8)** | **No dose  ramp-up**  **(*n=*8)** | **Conventional dose  ramp-up**  **(*n=*8)** | **Accelerated dose  ramp-up**  **(*n=*9)** | **No dose ramp-up (*n=*10)** |
| **Week 1** |  |  |  |  |  |  |
| Binding antibody | 2 (25.0) | 2 (25.0) | 2 (25.0) | 1 (12.5) | 1 (11.1) | 2 (20.0) |
| ***with titer ≥ 1:160*** | ***0*** | ***0*** | ***0*** | ***0*** | ***0*** | ***0*** |
| **Week 5** |  |  |  |  |  |  |
| Binding antibody | 2 (25.0) | 3 (37.5) | 2 (25.0) | 8 (100) | 4 (44.4) | 4 (40.0) |
| ***with titer ≥ 1:160*** | ***0*** | ***0*** | ***0*** | ***0*** | ***0*** | ***0*** |
| **Week 8** |  |  |  |  |  |  |
| Binding antibody | 2 (25.0) |  |  | 6 (75.0) |  |  |
| ***with titer ≥ 1:160*** | ***0*** |  |  | ***0*** |  |  |
| **Week 9** |  |  |  |  |  |  |
| Binding antibody |  | 3 (37.5) | 2 (25.0) |  | 5 (55.6) | 2 (20.0) |
| ***with titer ≥ 1:160*** |  | ***0*** | ***0*** |  | ***0*** | ***0*** |
| **End of study/early termination** |  |  |  |  |  |  |
| Binding antibody | 2 (25.0) | 2 (25.0) | 1 (12.5) | 3 (37.5) | 1 (11.1) | 2 (20.0) |
| ***with titer ≥ 1:160*** | ***0*** | ***0*** | ***0*** | ***0*** | ***0*** | ***0*** |

Assays in Weeks 8 and 9 correspond to the last doses in the treatment period, which were administered in Week 8 for the conventional ramp-up arms and Week 9 for the accelerated and no dose ramp-up arms

*rHuPH20*, recombinant human hyaluronidase; *TDL*, target dose level
